# Cancer cachexia and skeletal muscle atrophy in clinical studies: what do we really know?

**DOI:** 10.1002/jcsm.12633

**Published:** 2020-10-14

**Authors:** Adeline Dolly, Jean‐François Dumas, Stéphane Servais

**Affiliations:** ^1^ INSERM UMR 1069, Nutrition Croissance et Cancer Université de Tours Tours France

**Keywords:** Cancer cachexia, Skeletal muscle alterations, Clinical studies, Myosteatosis, Mitochondria

## Abstract

Research investigators have shown a growing interest in investigating alterations underlying skeletal muscle wasting in patients with cancer. However, skeletal muscle dysfunctions associated with cancer cachexia have mainly been studied in preclinical models. In the present review, we summarize the results of clinical studies in which skeletal muscle biopsies were collected from cachectic vs. non‐cachectic cancer patients. Most of these studies suggest the presence of significant physiological alterations in skeletal muscle from cachectic cancer patients. We suggest a hypothesis, which connects structural and metabolic parameters that may, at least in part, be responsible for the skeletal muscle atrophy characteristic of cancer cachexia. Finally, we discuss the importance of a better standardization of the diagnostic criteria for cancer cachexia, as well as the requirement for additional clinical studies to improve the robustness of these conclusions.

## Introduction

Cancer cachexia is a complex multifactorial syndrome characterized by involuntary and pathological weight loss, mainly due to skeletal muscle wasting. Patients experience a deterioration of their nutritional status, which is associated with a profound weakening of the body. Formation of oedemas, loss of appetite (or anorexia), and persistent fatigue are also observed. These effects considerably reduce patients' quality of life and overall survival. Cancer cachexia is a cause of death for 20–25% of patients,^1^ and it is also a co‐morbidity significantly affecting their overall survival. In 2011, a consensus of international experts defined cancer cachexia as being characterized by a progressive loss of skeletal muscle mass, with or without loss of adipose mass. This cachexia cannot be fully supported by conventional nutritional support and gradually leads to functional impairment.^2^ It has been described as a continuum with three stages of diagnosis: pre‐cachexia, cachexia, and refractory cachexia. The risk of progression depends on the stage and the type of tumour, the presence of systemic inflammation, the response to antitumour treatment, as well as inter‐individual variations such as genetic predisposition, body composition, food intake, physical activity, and co‐morbidities.^3^, ^4^ It is however important to note that the severity of cachexia does not appear to correlate with the size of the tumour.^5^ The prevalence of cachexia in cancer patients is estimated to be ~35%.^6^ It can even reach ~80–90% for gastric and pancreatic cancers, and it is particularly prevalent in advanced stages of cancer.^7^, ^8^

Because of the pathophysiological complexity and multifactorial characteristics of this clinical syndrome, there is currently no effective treatment for cancer cachexia. Current research have focused on a multimodal approach,^9^ which includes adapted anticancer treatment^10^, ^11^, ^12^; pharmacological treatment that aims in particular at reducing systemic inflammation, counteracting the hypercatabolic state of patients, and/or stimulating their appetite^13^, ^14^; nutrition care^15^, ^16^, ^17^, ^18^, ^19^, ^20^, ^21^; adapted physical activity^22^, ^23^, ^24^; and psychosocial care.^25^, ^26^ It is therefore essential to further improve our understanding regarding the interplay of the molecular mechanisms involved in the onset and progression of cancer cachexia. In particular, it is of crucial importance to better understand the pathophysiological basis of skeletal muscle atrophy, which represents a major clinical feature of cachexia.

To improve our knowledge of the factors regulating skeletal muscle mass loss, clinical studies are essential. The critical point for clinical studies investigating cancer cachexia is to identify cachectic vs. non‐cachectic cancer patients groups. The clinical definition established by Fearon *et al*. distinguishes between cachectic patients and non‐cachectic patients based on weight loss and body mass index.^2^ This definition has subsequently been validated in an international multicentre study with 861 patients.^27^ However, recent studies have demonstrated the importance of continually developing and updating these clinical criteria, which take into account the evolution of the overweight and obesity prevalence^28^ or the advanced age of most cachectic patients.^29^

The aim of this review was to offer an up‐to‐date synthesis of the data, which suggest the presence of alterations in skeletal muscle from cachectic cancer patients. In addition, we discuss the importance of a standardization for the diagnostic criteria of cancer cachexia, as well as the need for additional clinical studies to improve the robustness of the conclusions.

## Methodology

Articles indexed in PubMed were queried to identify clinical studies, which have analysed skeletal muscle from cachectic cancer patients. Search terms included ‘[(cancer) OR (carcinoma) OR (tumor) OR (malignant) OR (metastasis)] AND [(cachexia) OR (sarcopenia) OR (weight loss) OR (malnutrition)] AND [(skeletal muscle) OR (muscle mass) OR (lean body mass) OR (rectus abdominis) OR (quadriceps)] ± [(biopsy) OR (biopsies)]’ ± filters: humans; adult: 19 + years; and publication dates from 01/01/1900 to 18/02/2020 were included. Among 749 publications identified through the database search, we excluded duplicates, review articles, preclinical studies, non‐relevant or non‐full‐text clinical studies, as well as clinical studies where cachectic cancer patients were compared with healthy control patients.^30^, ^31^, ^32^, ^33^, ^34^, ^35^, ^36^, ^37^, ^38^, ^39^, ^40^, ^41^, ^42^, ^43^ Importantly, in that case, it is not possible to distinguish between the specific effects of cachexia and cancer on the various parameters examined. We also excluded a case study, which was carried out on a cachectic cancer patient without comparison with a population of non‐cachectic patients.^44^ The identified articles were manually searched to identify additional relevant publications.

Thirty‐one clinical studies were identified by this review of the literature (*Table* *1*). The vast majority of these studies discuss patients with gastrointestinal cancer, including cancer of the oesophagus, stomach, liver, pancreas, colon, and anus. The main mechanisms studied cover the structure and typing of muscle fibres, pathways involved in proteolysis and protein synthesis, as well as lipid and mitochondrial metabolisms.

**Table 1:** Clinical studies on skeletal muscle alterations associated with cancer cachexia

Clinical study	Cancer	Population	Diagnostic criteria for CC	Muscle iopsy	Observations upon comparing CC vs. CNC patients
Judge *et al*.^45^	Pancreas	Healthy *N* = 16 (31% ♂)	WL > 5% in 6 months	RA	Increased fibrotic tissue and collagen content, which positively correlated with WL percentage (*P* = 0.0016, *r* = 0.672). No difference in fat deposition and no significant correlation with WL percentage.
	Stages I–III	Cancer *N* = 20 (50% ♂)			
		CC: 75% (NI% ♂)			
Zhang *et al*.^46^	Stomach	Cancer *N* = 39 (72% ♂)	WL > 5% in 6 months ± muscle loss	RA	Significant decrease in myofibers cross‐sectional area in patients with muscle loss ± cachexia (*P* < 0.05). No difference for patients without muscle loss. Ultrastructure disorganization and autophagosome formation in CC patients. Increased protein and mRNA expression of autophagic–lysosomal (Beclin‐1, LC3B, and p62) and ubiquitin–proteasome (MuRF1 and polyubiquitinylated proteins) systems markers in CC patients (all *P* < 0.05).
	Stages I–III	CC: 56% (68% ♂)			
Ebhardt *et al*.^47^	Oesophagus, stomach, pancreas	Healthy *N* = 18 (56% ♂)	WL > 5%	Q	Comparison of proteomic signatures. In CC patients, reduced expression of TPM1, 2 and greater expression of MyHC1, 4, 8 (muscle contraction) compared with CNC. Deregulation of proteins in the mitochondrial electron transport chain and focal adhesion.
	Stages NI	Cancer *N* = 19 (79% ♂)			
		CC: 26% (60% ♂)			
Schmitt *et al*.^48^	Pancreas	Healthy *N* = 3	WL > 10% in 6 months	RA	Significant decrease in protein expression of MyHC (*P* = 0.036), actin, Akt (*P* = 0.001), FOXO1 (*P* = 0.011), phosphorylated forms of FOXO3a (*P* = 0.011), mTOR (*P* = 0.007), and S6K (*P* = 0.033) in CC vs. CNC patients. Regulators of muscle contraction, protein synthesis, and protein degradation inhibitors.
	Stages II and IV	Cancer *N* = 13			
		CC: 62% (38% ♂)			
Stephens *et al*.^49^	Upper gastrointestinal	Healthy *N* = 15 (53% ♂)	WL ≥ 5%	RA	Increased protein expression of β‐dystroglycan (muscle structure) in CC vs. CNC patients. No modification of protein expression levels of MyHC, β‐sarcoglycan, dystrophin (muscle structure), Akt, FOXO, MuRF1, MAFBx (muscle proteolysis), BNIP3, and GABARAPL1 (autophagy).
	Stages I–IV	Cancer *N* = 92 (72% ♂)			
		CC: 55% (63% ♂)			
Skorokhod *et al*.^50^	Pancreas	Cancer *N* = 23 (61% ♂)	WL > 10%	RA	Identification of 183 genes associated (positively for most of them) with CC. Some are involved in muscle contraction, rearrangement of the actin cytoskeleton, proteolysis, tissue hypoxia, and inflammatory response systems (e.g. over‐regulated Egr‐1).
	Stages II–IV	CC: 43% (50% ♂)			
Johns *et al*.^51^	Upper gastrointestinal, Pancreas	Cancer *N* = 41 (73% ♂)	WL > 5% ① or 10% ② in 6 months or >2% + muscle loss ③	RA	Significant decrease in mean muscle fibre diameter in patients with muscle loss ± WL (~25%, *P* = 0.001 and *P* = 0.02, respectively) compared with CNC patients. No difference for patients without muscle loss. No difference in fibre number or proportion of fibre type across all MyHC isoforms. Decrease in mean protein content and RNA/DNA ratio in patients with >5% WL or >2% WL + muscle loss compared with CNC patients. Increase in SMAD3 (*P* = 0.022—atrophy), beclin, and ATG5 (autophagy—*P* = 0.05 and 0.01, respectively) protein levels in patients with WL compared with CNC patients. No difference in ATG7 (autophagy), phospho‐NF‐κB, or phospho‐STAT3 (inflammatory cytokine signalling) protein levels.
		CC: 44% (61% ♂)			
		①			
	Stages NI	or 27% (64% ♂) ②			
		or 41% (65% ♂) ③			
Op den Kamp *et al*.^52^	NSCLC	Healthy *N* = 22 (59% ♂)	International consensus from 2011^2^	Q	No difference observed in the cross‐sectional area of muscle fibres, protein concentration per unit of DNA, and muscle strength. No difference observed in the proteins of the Akt/FOXO pathway and of the UPS (including MuRF1 and MAFBx). Regarding the autophagic–lysosomal system, increased protein expression of BNIP3 in CC vs. CNC patients, no difference observed for LC3B. No difference observed for luciferase activity of NF‐κB, and for mRNA expression of IκBα (NF‐κB inhibitor).
	Stages IIIb/IV	Cancer *N* = 26 (65% ♂)			
		CC: 62% (56% ♂)			
Taskin *et al*.^53^	Gastrointestinal	Healthy *N* = 5 (40% ♂)	WL > 10% in 6 months	RA	Calcium sensitivity of the contractile apparatus is significantly increased in CC vs. CNC patients. No difference in absolute strength or ubiquitin protein polymers. Insignificant trend in a decrease in the ratio of MyHC I: IIa isoforms, indicating a higher number of fast muscle fibres in CC vs. CNC patients.
	Stages NI	Cancer *N* = 14 (50% ♂)			
		CC: 43% (33% ♂)			
Op den Kamp *et al*.^54^	NSCLC	Healthy *N* = 22 (59% ♂)	International consensus from 2011 ^2^	Q	No difference observed in fibre typing, oxidative, and glycolytic enzymatic activities, protein expression of mitochondrial respiratory chain complexes, and markers of mitochondrial biogenesis (PGC1α and TFAM).
	Stages IIIb/IV	Cancer *N* = 26 (65% ♂)			
		CC: 62% (56% ♂)			
de Castro *et al*.^55^	Stomach, colorectal	Cancer *N* = 44 (55% ♂)	WL > 5% in 12 months maximum^56^ + C‐reactive protein + Glasgow score + Cachexia Symptoms Questionnaires	RA	Increased mRNA expression of Fis1 (mitochondrial fission) (*P* = 0.03). No modification of the transcripts of Mfn2 (mitochondrial fusion), TFAM, and PGC1α (mitochondrial biogenesis). Increase in the intermyofibrillary mitochondrial area in TEM (*P* = 0.01). No change in the number of copies of mitochondrial DNA. Increased protein expression of LC3II (*P* = 0.02), ATG5 (*P* = 0.042), and ATG7 (*P* = 0.03) (autophagy). Increased protein expression of activated caspases 8 (*P* = 0.037) and 9 (*P* = 0.046) and phosphorylated p53 (*P* = 0.041) (apoptosis).
	Stages I–IV	CC: 55% (67% ♂)			
Aversa *et al*.^57^	Several cancers	Healthy *N* = 11 (64% ♂)	WL > 5% in 6 months	RA	Increased mRNA expression of LC3B and protein expression of LC3BII and Parkin, decreased mRNA expression of Parkin and PINK1 in CC vs. CNC patients (*P* < 0.05). No change regarding Beclin‐1, p62, BNIP3, and Nix (autophagy/mitophagy markers).
	Stages I–IV	Cancer *N* = 29 (59% ♂)			
		CC: 41%			
Bossola *et al*.^58^	Stomach	Healthy *N* = 14 (64% ♂)	None	RA	The specific chymotrypsin activity of the proteasome is increased when WL is ≥10% (*P* = 0.003). No change in trypsin and peptidyl‐glutamyl‐peptidase activities.
	Stages I–IV	Cancer *N* = 23 (61% ♂)			
Khal *et al*.^59^	Colorectum, pancreas	Healthy *N* = 10 (80% ♂)	WL moderate > 1%, severe > 11%	RA	Increase in mRNA and protein expression of proteasome 20S subunits and protein expression of E214k (ubiquitination), depending on WL (especially when it is between 10% and 20%).
	Stages NI	Cancer *N* = 18 (67% ♂)			
		CC: 72% (69% ♂)			
Narasimhan *et al*.^60^	Pancreas, colorectum	Cancer *N* = 40 (43% ♂)	International consensus from 2011^2^	RA	Differential expression of 922 genes subjected to alternative splicing (772 up‐regulated and 150 down‐regulated) in CC vs. CNC patients. Genes involved in myogenesis, lipid biosynthesis, protein ubiquitination (and proteolysis) and inflammation associated positively, for the vast majority of them, with CC.
	Stages I–IV	CC: 53% (38% ♂)			
MacDonald *et al*.^61^	Upper gastrointestinal	Healthy *N* = 7 (42% ♂)	WL ≥ 5%	RA + Q	Increased protein synthesis in CC vs. CNC patients (0.073% vs. 0.061% per hour, *P* = 0.022). Higher in Q vs. RA in CC (*P* = 0.021). No difference in proteolysis.
	Stages I–IV	Cancer *N* = 14 (57% ♂)			
		CC: 57% (50% ♂)			
D'Orlando *et al*.^62^	Stomach	Healthy *N* = 12 (58% ♂)	WL > 5% in 6 months	RA	No difference in mRNA expression of muscle atrophy genes (Atrogin‐1, MuRF1, myostatin, and follistatin).
	Stages I–IV	Cancer *N* = 38 (66% ♂)			
		CC: 18%			
Rhoads *et al*.^63^	Stomach	Healthy *N* = 10 (60% ♂)	None	RA	A more or less significant WL does not modify protein expression of IκBα (NF‐κB inhibitor).
	Stages Ib–IV	Cancer *N* = 14 (57% ♂)			
Stephens *et al*.^64^	Upper gastrointestinal	Healthy *N* = 7 (71% ♂)	WL ≥ 5%	RA + Q + D	Significant association between the mRNA expression of two genes activated by exercise and WL: CaMKIIβ in the RA (*r* = 0.82, *P* = 0.01), Q (*r* = 0.45, *P* = 0.06), and the D (*r* = 0.50, *P* = 0.03) and TIE1 in the RA (*r* = 0.67, *P* = 0.01) and Q (*r* = 0.70, *P* = 0.003). No correlation observed for E3 ubiquitin ligases and proteins of the Akt/FOXO pathway.
	Stages NI	Cancer *N* = 65 (71% ♂)			
		CC: NI			
Eley *et al*.^65^	Stomach, oesophagus	Healthy *N* = 9 (10% ♂)	None	RA	No correlation between WL and the protein expression of the phosphorylated forms of PKR and eIF2α (factors of inhibition of protein synthesis). The higher the WL, the lower the protein expression of myosin.
	Stages I–IV	Cancer *N* = 15 (87% ♂)			
Bossola *et al*.^66^	Stomach	Healthy *N* = 10 (60% ♂)	None	RA	WL severity does not influence the mRNA expression of ubiquitin.
	Stages I–IV	Cancer *N* = 20 (55% ♂)			
Bossola *et al*.^67^	Stomach	Healthy *N* = 5 (60% ♂)	WL moderate > 5%, severe > 10%	RA	No difference in the percentage of apoptotic nuclei between patients with mild or moderate–severe weight loss.
	Stages I‐IV	Cancer *N* = 16 (50% ♂)			
		WL > 5%: 69% (45% ♂)			
Stephens *et al*.^68^	Oesophagus, stomach, pancreas + others	Healthy *N* = 6 (33% ♂)	None	RA	In cancer patients, the greater the WL, the more the number of intramyocellular lipid droplets increases (*r* = 0.51, *P* = 0.025). No correlation with the diameter of the lipid droplets.
	Stages II–IV	Cancer *N* = 19 (58% ♂)			
Johns *et al*.^69^	Several cancers	Total *N* = 1276 (61% ♂)	WL > 5% or 10% or 15% or low SMI + WL > 2%	RA	Depending on the diagnostic criteria for CC, the SNPs associated with CC as well as their degree of association (*P* value) differ. When WL > 10%, the highlighted SNPs participate in the regulation of appetite (*P* = 0.004), adhesion (*P* = 0.005), structure and function of the cell membrane (*P* = 0.037), and signal transduction (*P* = 0.038). When WL > 15%, they only participate in cell adhesion (*P* = 0.019). When WL > 2% and the SMI is low, they participate in the regulation of appetite (*P* = 0.014), signal transduction (*P* = 0.023), glucocorticoid‐regulated pathway (*P* = 0.034), and in lipid metabolism (*P* = 0.039).
	Stages I–IV	CC: NI			
Narasimhan *et al*.^70^	Pancreas, colorectum	Cancer *N* = 42 (43% ♂)	International consensus from 2011^2^	RA	Identification of eight new miRNAs associated with CC and participating in particular in pathways regulating lipid biosynthesis, myogenesis, inflammation, and the innate immune response.
	Stages I–IV	CC: 52% (41% ♂)			
Marzetti *et al*.^71^	Stomach	Healthy *N* = 9 (89% ♂)	WL > 5% in 6 months	RA	No difference in protein expression of Mfn2, OPA1 (mitochondrial fusion), PINK1, and Parkin (mitophagy) or in mRNA expression of PGC1α and TFAM (mitochondrial biogenesis). mRNA expression of Fis1 (mitochondrial fission) and protein expression of LC3BI (autophagy) increased in CC vs. CNC patients (*P* < 0.05).
	Stages I‐IV	Cancer *N* = 18 (94% ♂)			
		CC: 50% (89% ♂)			
Collins *et al*.^72^	Gastrointestinal	Healthy *N* = 6 (33% ♂)	WL	RA	Increased mRNA expression of UCP‐3 in weight‐losing cancer patients compared with weight‐stable cancer patients (*P* < 0.02). No difference in mRNA expression of UCP‐2.
	Stages NI	Cancer *N* = 12 (92% ♂)			
		CC: 50% (100% ♂)			
Brzeszczyńska *et al*.^73^	Oesophagus, stomach, pancreas	Healthy *N* = 41 (80% ♂)	WL > 5%	Q	No difference in mRNA expression of satellite cell markers (Pax3 and Pax7), early (MyoD and Myf5) and late myogenesis (MyoG), autophagic factor (p62), and antioxidant defence genes (SOD2, GCLM, NSF2, and HSP1a).
	Stages II–III	Cancer *N* = 28 (75% ♂)			
		CC: 36% (80% ♂)			
Prokopchuk *et al*.^74^	Pancreas	Healthy N = 19 (37% ♂)	WL > 10% in 6 months	RA	No difference in mRNA expression of IL‐4, IL‐4R, and IL‐13R (inflammatory markers).
	Stages I–IV	Cancer *N* = 25 (32% ♂)			
		CC: 48% (25% ♂)			
Ramage *et al*.^75^	Upper gastrointestinal	Cancer *N* = 32 (81% ♂)	WL > 5%	RA	Positive correlation between protein content and skeletal muscle radiodensity on CT scan (*r* = 0.406, *P* = 0.021), as well as WL (*r* = 0.416, *P* = 0.018).
	Stages I–IV	CC: 47%			
Sun *et al*.^76^	Stomach	Healthy N = 29 (72% ♂)	None	RA	Significant association between mRNA expression of ubiquitin and TRAF6 with WL (*P* = 0.001 for both).
	Stages I–IV	Cancer *N* = 102 (71% ♂)			

Akt or PKB, protein kinase B; ATG5/7, autophagy‐related 5/7; BNIP3, BCL2 interacting protein‐3; CaMKIIβ, calcium/calmodulin‐dependent protein kinase‐IIβ; CC, cancer cachectic; CNC, cancer non‐cachectic; CT, computed tomography; D, diaphragm muscle; E214k, 14 kDa ubiquitin‐conjugating enzyme; eIF2α, eukaryotic initiation factor‐2α; Egr‐1, early growth response protein‐1; Fis1, mitochondrial fission protein‐1; FOXO, forkhead box O protein; GABARAPL1, GABA type A receptor associated protein like‐1; GCLM, glutamate‐cysteine ligase modifier subunit; HSP1, heat shock 70 kDa protein‐1; IκBα, nuclear factor of kappa light polypeptide gene enhancer in B‐cells inhibitor‐α; IL, interleukin; LC3B or MAP 1‐LC3B, microtubule‐associated protein 1‐light chain 3 beta; MAFBx or atrogin‐1, muscle atrophy F‐box; Mfn, mitofusin; mTOR, mechanistic target of rapamycin; MuRF1, muscle RING finger‐1; MyHC, myosin heavy chains; MyoD, myoblast determination protein‐1; Myf5, myogenic factor‐5; MyoG, myogenin; NF‐κB, nuclear factor of kappa light polypeptide gene enhancer in B‐cells; NI, not indicated; Nix or BNIP3‐like, NRF2, or NFE2L2, nuclear factor (erythroid‐derived 2)‐like 2; NSCLC, non‐small‐cell lung cancer; OPA1, optic atrophy type 1; Pax, paired box; PGC1α, peroxisome proliferator‐activated receptor gamma coactivator 1‐α; PINK1, PTEN‐induced kinase‐1; PKR, protein kinase R; Q, quadriceps; RA, *rectus abdominis*; S6K1, protein S6 kinase 1; SMAD3, mothers against decapentaplegic homologue 3; SMI, skeletal muscle index; SNP, single nucleotide polymorphism; SOD2, superoxide dismutase‐2; STAT3, signal transducer and activator of transcription 3; TEM, transmission electron microscopy; TFAM, mitochondrial transcription factor A; TIE1, tyrosine kinase with immunoglobulin‐like and EGF‐like domains‐1; TPM, tropomyosin; TRAF6, tumour necrosis factor receptor (TNFR)‐associated factor‐6; UCP, uncoupling protein; UPS, ubiquitin–proteasome system; WL, weight loss.

## Skeletal muscle alterations in cancer cachexia

### Structure and typing of muscle fibres

Up until now, cancer cachexia has mainly been studied in preclinical models generally characterized by transplantation of cancer cells or injection of carcinogens. The most studied and best described models are colon‐26 adenocarcinoma^77^ and Lewis lung adenocarcinoma.^78^ In these models, the cross‐sectional area of muscle fibres decreases,^79^ and this atrophy is more likely to affect type II fibres.^80^

In clinical studies, little is known about the possible alterations in the structure and typing of muscle fibres in patients with cancer cachexia (*Table* *1*). Judge *et al*. have reported increased fibrosis and collagen content in skeletal muscle from cachectic pancreatic cancer patients.^45^ Skeletal muscle ultrastructure also appears to be impaired, with an apparent disorganization and autophagosome formation in gastric cancer patients with cachexia.^46^ The protein expression of myosin heavy chains, a major component of the muscular contractile system, was increased (for isoforms 1, 4, and 8),^47^ decreased,^48^ or unchanged^49^ in cachectic patients with gastrointestinal cancer. Other muscular structural components have also been studied. The protein expression of actin and tropomyosin 1 and 2 was shown to be reduced^47^, ^48^ while that of β‐dystroglycan was increased, and those of β‐sarcoglycan and dystrophin remained unchanged^49^ in cachectic patients with gastrointestinal cancer. Skorokhod *et al*. also identified genes associated (positively for most of them) with cancer cachexia.^50^ These genes are involved in muscle contraction and development (e.g. actin, titin, tropomyosin, and troponin) and actin cytoskeleton rearrangement (e.g. cofilin, dystonin, and vinculin) in pancreatic cancer patients.^50^ While a significant reduction in the cross‐sectional area of muscle fibres was observed in gastrointestinal cancer patients with cachexia and muscle loss,^46^, ^51^ Op den Kamp *et al*. found no such change in cachectic patients with advanced non‐small‐cell lung cancer compared with pre‐cachectic patients.^52^ Finally, no significant alteration in fibre typing associated with cancer cachexia has been reported in clinical studies, either of gastrointestinal^51^, ^53^ or lung^54^ cancer.

### Muscle proteolysis and protein synthesis

In preclinical studies of cancer cachexia, there is abundant evidence for increased proteolysis, particularly through autophagy and the ubiquitin–proteasome system (UPS), and decreased protein synthesis in skeletal muscle (see review^81^, ^82^, ^83^). Activation of NF‐κB by inflammatory cytokines may result in muscle wasting in mouse models.^84^ NF‐κB induces the transcription of UPS genes, which activation has also been observed in preclinical models.^82^, ^83^ Apoptotic processes may also, at least in part, be responsible for muscle atrophy, as suggested by the data from different preclinical studies.^85^, ^86^, ^87^, ^88^

However, results from clinical studies are much more controversial (*Table* *1*). Some studies have reported an increased expression of autophagy markers (Beclin‐1, LC3B, ATG5, ATG7, and p62)^46^, ^51^, ^55^, ^57^ and of UPS proteins (MuRF1 and polyubiquitinylated proteins),^46^ as well as muscle atrophy‐inducing pathway regulators (SMAD3)^51^ in cachectic patients with mainly gastrointestinal cancer. Increased proteasome activity in gastric cancer^58^ and increased protein expression of proteasome 20S subunits in colorectal and pancreatic cancers^59^ were also reported. Narasimhan *et al*. and Skorokhod *et al*. have identified many genes positively (e.g. protein degradation and ubiquitination genes) associated with cancer cachexia in pancreatic and/or colorectal cancer patients.^50^, ^60^ Schmitt *et al*. have observed a decrease in the expression of actors involved in protein synthesis and inhibition of protein degradation pathways (Akt, FOXO, mTOR, and S6K) in pancreatic cancer.^48^ However, numerous clinical studies have found no impairment in the signalling pathways involved in the regulation of proteolysis and protein synthesis in skeletal muscle from cachectic patients.^49^, ^52^, ^53^, ^61^, ^62^ In many cases, no association with weight loss was observed^63^, ^64^, ^65^, ^66^ in gastrointestinal and pulmonary cancers. Johns *et al*. reported no difference in the levels of muscle atrophy inflammatory mediators phospho‐NF‐κB and phospho‐STAT3 protein levels in gastrointestinal cancer patients with cachexia.^51^ Regarding apoptotic pathways, de Castro *et al*. have observed an increased expression of several well‐known markers (activated caspases 8 and 9 and phosphorylated p53) in gastrointestinal cancer patients with cachexia.^55^ However, Bossola *et al*. have reported unchanged number of apoptotic myonuclei when comparing gastric cancer patients with mild or moderate–severe weight loss.^67^

### Myosteatosis

Myosteatosis, which is defined as a pathological fat accumulation in skeletal muscle, has been studied in a preclinical tumour‐bearing model (Ward colon tumour model).^89^ In that case, the authors observed increased neutral lipids and total triglyceride content within the rat *gastrocnemius* muscle fibres. This observation is indicative of the presence of lipid droplets, as well as increased mRNA levels of key transcription factors involved in adipocyte gene expression (i.e. C/EBPδ, C/EBPα, and PPARγ).

In cachectic cancer patients, weight loss has been associated with a greater number of intramyocellular lipid droplets, as observed by electron microscopy of abdominal muscle biopsies.^68^ In the clinical practice, studies of skeletal muscle radiological attenuation by computed tomography scan have shown that it is very variable in cancer patients. Its decrease may, at least in part, reflect an accumulation of intramuscular lipids^90^, ^91^ and is associated with shorter survival.^92^ The specific pathophysiological mechanisms that lead to myosteatosis have not yet been well‐characterized. Transcriptomic analyses have suggested that disruption in oxidative phosphorylation and lipid accumulation may contribute to myosteatosis, as observed in abdominal skeletal muscle biopsies of patients with pancreatic or periampullary cancer and with significantly low radiological attenuation of skeletal muscle on computed tomography scan.^93^ Genetic and transcriptomic studies of skeletal muscle biopsies obtained from cachectic patients (mainly gastrointestinal cancers) have identified single nucleotide polymorphism^69^ and miRNA^70^ involved in lipid biosynthesis (*Table* *1*). Narasimhan *et al*. also found alternatively spliced genes, which are mostly up‐regulated, and encode enzymes and binding proteins from the lipid biosynthesis pathways.^60^

### Mitochondrial metabolism

Previous studies have suggested an association between mitochondria dysfunctions and skeletal muscle atrophy.^94^, ^95^ Several preclinical and clinical studies have therefore investigated mitochondrial metabolism dysregulation in skeletal muscle in the context of cancer cachexia. Muscle atrophy associated with mitochondrial dysfunctions has been observed in cachectic rodents. Among these dysfunctions, several parameters have been described, and they include increased mitochondrial surface^96^, ^97^; impairment of mitochondrial dynamics such as increased fission (Fis1), decreased fusion (Mfn1 and Mfn2), or biogenesis (PGC1α)^98^; a decrease in the activity of the respiratory chain complexes^99^, ^100^, ^101^; and an increase in UCP‐2 and UCP‐3 gene expression,^102^, ^103^ a questionable indicator of mitochondrial energy coupling. The study by Brown *et al*. on preclinical models of cancer cachexia suggested that alterations in dynamics (biogenesis and fusion), mitochondrial quality, and function precede muscle atrophy and that a decrease in mitochondrial protein content as well as an increase in mitophagy appears at a later stage of cachexia.^104^

Regarding clinical studies, mitochondrial metabolism has rarely been studied in the context of cancer cachexia (*Table* *1*). A recent clinical study by de Castro *et al*., including patients with gastric or colorectal cancer, revealed an increase in Fis1 mRNA expression in skeletal muscle from cachectic patients compared with non‐cachectic, but there was no modification in the levels of the fusion marker (Mfn2) and mitochondrial biogenesis transcripts (TFAM and PGC1α).^55^ On the other hand, they showed by electron microscopy that there was an increase in the intermyofibrillary mitochondrial area, without modification in the number of mitochondrial DNA copies. This finding could illustrate the absence of modification in mitophagy activity. The authors suggested that these last observations supported the results from the preclinical study by Brown *et al*. who showed that impaired mitochondrial protein content and increased mitophagy only appear at a much later stage in cancer cachexia.^104^ Similarly, Marzetti *et al*. have observed an increase in Fis1 transcript levels and no change in mRNA or protein expression levels of markers of mitochondrial fusion (Mfn2 and OPA1) and mitochondrial biogenesis (PGC1α and TFAM) in cachectic patients with gastric cancer.^71^ Op den Kamp *et al*. have also reported an absence of modification of the protein expression of the same markers of mitochondrial biogenesis (PGC1α and TFAM), as well as complexes of the respiratory chain in cachectic patients suffering from pulmonary cancer.^54^ On the other hand, Collins *et al*. have observed increased UCP‐3 (but not UCP‐2) mRNA expression levels in patients with gastrointestinal adenocarcinoma experiencing weight loss.^72^ This finding suggests that increased proton leak may contribute to skeletal muscle catabolism through enhancement of energy expenditure. However, increase in skeletal muscle UCP‐3 mRNA is a poor index of mitochondrial energy coupling. Finally, to our knowledge, it is important to note that no clinical study has explored the mitochondrial functionality in skeletal muscle from cachectic cancer patients.

## Discussion and other perspectives

The overall result of clinical studies suggests the presence of alterations in skeletal muscle from cachectic cancer patients (*Table* *1*). These alterations may affect the structure of muscle fibres, the different pathways involved in proteolysis and protein synthesis, lipid metabolism (myosteatosis), and mitochondrial metabolism (i.e. mitochondrial surface and dynamics and mitochondrial DNA).

It is important to note that for most of the parameters studied, which could play a role in muscle wasting, the results of clinical studies diverge (*Table* *1*). A possible explanation for these differences may be the lack of well‐established diagnostic criteria. An improvement and better standardization of the diagnostic criteria used for cancer cachexia identification is utterly necessary. These criteria should include key cachexia symptoms such as weight loss, taking body mass index into account,^28^ muscle wasting,^105^, ^106^ appetite loss, performance status, and blood chemistry. On the basis of these key components, Zhou *et al*. have proposed a clinically applicable score for the classification of the various cachexia stages observed in cancer patients.^107^ De Castro *et al*. have also used combined diagnostic criteria to discriminate cachectic and non‐cachectic cancer patients.^55^ To better improve patient care, it would be preferable to diagnose patients as soon as possible, possibly at a pre‐cachectic state. Early diagnosis can reduce the risk of transition to a cachectic state with an appropriate choice of therapeutic and supportive regimens. But current diagnostic criteria are insufficient and inadequate to allow a good distinction between pre‐cachectic and non‐cachectic patients.^27^ New clinical trials testing muscle bioptic and biochemical/metabolic parameters are necessary to develop validated criteria.

Besides diagnostic criteria, more obvious factors may explain why the results of clinical studies on skeletal muscle alterations in cancer cachexia diverge (*Table* *1*): (i) The heterogeneity of the cohorts, sometimes with several types of cancer and a disproportionate distribution of these types between the cachectic and non‐cachectic groups. At this time, we cannot exclude that each cancer can differently regulate cancer cachexia. Additionally, there is a disproportionate distribution of men and women between groups. Sexual dimorphism exists for several skeletal muscle parameters, and hormonal differences may also have a significant impact on cancer cachexia pathophysiology.^108^, ^109^, ^110^ (ii) Muscular localization of biopsies. Most samples were collected from the *rectus abdominis*, but some clinical studies have used biopsies from the *quadriceps* ^47^, ^52^, ^54^, ^61^, ^64^, ^73^ or diaphragm muscle.^64^ (iii) The number of patients included is often very small and cannot provide sufficient statistical power. Thirteen of 31 clinical studies had a total number of cancer patients ≤20, and there were <30 patients in 20 studies, with a prevalence of cachexia of around 50% most of the time. (iv) Methods of analysis. Some studies have relied on mRNA expression, while others have compared protein expression or enzyme activity. These differences may explain the varying results in clinical studies on muscle proteolysis.

Several blood components have been proposed to aid in the diagnosis of malnutrition/cancer cachexia in patients. Numerous recent clinical studies have observed an association between the components listed hereafter and the cachectic state of patients (*Table* *2*): (i) markers of inflammation: C‐reactive protein; interleukins IL‐1, IL‐6, IL‐8, IL‐10; IFN‐γ; and TNF‐α; (ii) members of the TGF‐β family: myostatin, activin A, and GDF‐15; (iii) factors derived from the tumour: ZAG (or LMF), VEGF, and Midkine; (iv) lipolysis markers of the adipose tissue: leptin, adiponectin, resistin, free fatty acids, and glycerol; and (v) others: ghrelin, IGF‐1, albumin, and angiotensin II. Other factors have also been studied, and they include the proteolysis‐inducing factor^121^ and markers of muscle degradation: 3‐methylhistidine, titin fragments, collagen fragments,^122^ testosterone,^123^ and the parathyroid hormone‐related protein.^124^ Nevertheless, there is currently not enough clinical evidence to conclude on their validity. To date, there is no validated biomarker for cancer cachexia.

**Table 2:** Recent clinical studies looking for potential biomarkers of cancer cachexia

Clinical study	Cancer, population	Diagnostic criteria for CC	Observations upon comparing CC vs. cancer non‐cachectic patients			
Stephens *et al*.^49^	Gastrointestinal	Weight loss ≥ 5%	C‐reactive protein, mg/L	18.3 (±32.6) vs. 12.0 (±29.6), *P* = ns		
	Stages I–IV					
	*N* = 92, CC: 55%					
Skorokhod *et al*.^50^	Pancreas	Weight loss > 10%	C‐reactive protein, NI	11.6 (3.1–25.6) vs. 7.3 (5.7–11.8), *P* = ns		
	Stages II–IV					
	*N* = 23, CC: 43%					
Aversa *et al*.^57^	Several cancers	Weight loss > 5% in 6 months	C‐reactive protein, mg/dL	2.07 (±0.98) vs. 1.92 (±1.04), *P* = ns	Albumin, g/dL	3.5 (±0.2) vs. 3.8 (±0.2), *P* = ns
	Stages I–IV					
	*N* = 29, CC: 52%					
Prokopchuk *et al*.^74^	Pancreas	Weight loss > 10% in 6 months	C‐reactive protein, mg/dL	0.3 (0.1–4.7) vs. 1.0 (0.1–4.0), *P* = ns	Albumin, g/dL	4.0 (3.7–4.4) vs. 4.0 (3.2–4.7), *P* = ns
	Stages I–IV					
	*N* = 25, CC: 48%					
Agustsson *et al*.^111^	Several cancers	Weight loss > 5% in 3 months or >10% in 6 months	C‐reactive protein, μg/L	26.9 (±11.6) vs. 4.9 (±2.2), *P* = ns	IGF‐1, μg/L	109.8 (±10.2) vs. 111.3 (±13.8), *P* = ns
	Stages I–IV		Leptin, ng/mL	↓ 4.5 (±0.6) vs. 9.0 (±1.5), *P* < 0.05	Free fatty acids, μmol/L/kg fat mass	↑ 53.8 (±8.6) vs. 32.5 (±3.6), *P* < 0.05
	*N* = 40, CC: 32.5%		Albumin, g/L	35.1 (±1.3) vs. 37.2 (±0.9), *P* = ns	Glycerol, μmol/L/kg fat mass	↑ 6.9 (±1.3) vs. 3.9 (±0.6), *P* < 0.05
Kim *et al*.^112^	Colorectal, lung	Weight loss ≥ 5% in 6 months	C‐reactive protein, mg/dL	3.0 (±4.0) vs. 1.6 (±2.3), *P* = 0.22	Albumin, g/dL	↓ 3.7 (±0.5) vs. 4.0 (±0.4), *P* = 0.04
	Stages I–IV					
	*N* = 42, CC: 50%					
Lerner *et al*.^113^	Several cancers	Not clearly specified. Maybe Fearon *et al*.^2^	IL‐1α, pg/mL	369.4 vs. 282.9, *P* = 0.809	IFN‐γ, pg/mL	217.8 vs. 211.5, *P* = 0.779
	Stages NI		IL‐1β, pg/mL	7.1 vs. 6.8, *P* = 0.913	TNF‐α, pg/mL	126.0 vs. 105.8, *P* = 0.665
	*N* = 92, CC: 53%		IL‐6, pg/mL	30.4 vs. 21.9, *P* = 0.117	VEGF, pg/mL	49.4 vs. 36.4, *P* = 0.286
			IL‐8, pg/mL	48.9 vs. 41.7, *P* = 0.764	Activin A, ng/mL	↑ 1.1 vs. 0.6, *P* = 0.028
			IL‐10, pg/mL	20.6 vs. 17.0, *P* = 0.374	GDF15, ng/mL	↑ 5.0 vs. 2.7, *P* = 0.025
Penafuerte *et al*.^114^	Several cancers	International consensus from 2011^2^	C‐reactive protein, mg/L	↑ ~21 vs. ~3, *P* < 0.0001	TGF‐β1, pg/mL	↑ ~22 000 vs. ~10 000, *P* < 0.0001
	Stages III–IV		IL‐6, pg/mL	↑ ~40 vs. ~15, *P* = 0.0054	Albumin, mg/L	↓ ~35 vs. ~41, *P* = 0.0027
	*N* = 122, CC: 50.8%		IL‐8, pg/mL	↑ ~60 vs. ~20, *P* = 0.001	Angiotensin II, pg/mL	↑ ~17 vs. ~8, *P* = 0.022
Loumaye *et al*.^115^	Colorectal, lung	International consensus from 2011^2^	C‐reactive protein, mg/dL	↑ 1.3 (0.1–25.7) vs. 0.3 (0.0–10.3), *P* < 0.001	Activine A, pg/mL	↑ 558 (228–17 660) vs. 397 (165–2731), *P* < 0.001
	Stage I–IV		Albumin, g/dL	↓ 4.2 (2.8–5.0) vs. 4.5 (3.0–5.1), *P* < 0.001	Myostatine, pg/mL	↓ 1371 (167–4989) vs. 2109 (715–4907), *P* < 0.001
	*N* = 152, CC: 49%					
Batista *et al*.^116^	Gastrointestinal	Weight loss > 5% in 3 months or >10% in 6 months	C‐reactive protein, μg/mL	↑ 24.9 (±14) vs. 14.9 (±13), *P* = 0.015	TNF‐α, pg/mL	↑ 72.5 (±29) vs. 13.8 (±4.3), *P* = 0.046
	Stages I–IV		IL‐6, pg/mL	↑ 160 (±58) vs. 30.3 (±8.2), *P* = 0.011	Leptin, pM	↓ 221 (±191) vs. 309 (±271), *P* = ?
	*N* = 43, CC: 72%		IL‐10, pg/mL	24.3 (±19) vs. 4.9 (±3.6), *P* = ns	Adiponectine, μg/mL	↑ 19.1 (±7.3) vs. 11.1 (±8.5), *P* = 0.010
Burney *et al*.^117^	Several cancers	IMC < 35 kg/m^2^	C‐reactive protein, mg/dL	↑ 3.5 (±1.1) vs. 1.8 (±0.8), *P* = 0.03	TNF‐α, pg/mL	5.0 (3–7.8) vs. 4.0 (2.9–6.2), *P* = ns
	Stages I–IV	Weight loss > 5% in 6 months				
	*N* = 95, CC: 47%					
Felix *et al*.^118^	Pancreas	Weight loss > 12% in 6 months	ZAG, μg/mL	↑ 40.3 (34.5–56.5) vs. 28.9 (23.8–30.3), *P* = 0.001		
	Stage II					
	*N* = 33, CC: 70%					
Kerem *et al*.^119^	Stomach	Perte d'IMC > 10% in 6 months	IL‐1β, pg/mL	0.03 (0–0.07) vs. 0 (0–0.05), *P* = ns	IFN‐γ, pg/mL	0.8 (0.2–1.6) vs. 0.5 (0.2–1.6), *P* = ns
	Stages I–IV		IL‐6, pg/mL	↑ 3.2 (1.4–6.2) vs. 2.1 (1–4.2), *P* = 0.005	Albumin, g/dL	↓ 3.4 (±0.1) vs. 3.8 (±0.07), *P* = 0.05
	*N* = 30, CC: 50%		IGF‐1, pg/mL	↓ 43.8 (±9.5) vs. 63.1 (±13.1), *P* < 0.001	Resistin, ng/mL	↑ 66.7 vs. 43.4, *P* < 0.001
			Leptin, pg/mL	↑ 3405 (±640) vs. 2623 (±665), *P* = 0.003		
Krzystek‐Korpacka *et al*.^120^	Stomach, oesophagus	Weight loss ≥ 5% in 3 months	IL‐1, pg/mL	2.8 (2.0–3.4) vs. 2.6 (2.0–3.1), *P* = 0.61	VEGF‐A, pg/mL	328 (232–378) vs. 227 (170–381), *P* = 0.130
	Stages II–IV		IL‐6, pg/mL	↑ 6.6 (3.5–10.0) vs. 3.0 (1.7–5.1), *P* = 0.005	VEGF‐C, ng/mL	16.0 (13.7–17.8) vs. 17.6 (15.9–19.8), *P* = 0.415
	*N* = 96, CC: 51%		IL‐8, pg/mL	↑ 47.8 (26.9–68.0) vs. 21.8 (18.0–35.4), *P* = 0.006	Midkine, pg/mL	1975 (1356–2397) vs. 1375 (1133–1945), *P* = 0.053
			TNF‐α, pg/mL	0.9 (0.4–2.6) vs. 1.5 (0.9–2.7), *P* = 0.207		

CC, cancer cachectic; NI, not indicated; ns, non‐significant.

In addition to variation in the levels of factors discussed before, heterogeneity in blood analysis data may also be due to the fact that concentrations of markers, such as IL6, TNFα, and leptin, may vary between day and intraday.^125^ These values may be partly associated to the patient's circadian rhythm and/or metabolic status.

It is interesting to note that C‐reactive protein does not appear to be modulated by circadian rhythm or metabolic status.^126^, ^127^ Despite this observation, levels of C‐reactive protein, which is a well‐accepted marker of systemic inflammation, are not consistently increased in populations of patients with cancer cachexia^49^, ^50^, ^57^, ^74^, ^111^, ^112^ (*Table* *2*). This observation challenges the use of C‐reactive protein in the clinic as a complementary biomarker for cancer cachexia diagnosis.

Considering the conflicting results obtained using muscle biopsies from cachectic patients, no specific biomarker of cancer cachexia has been identified at muscle levels. Myokines, such as myostatin, irisin, activin A, IL‐15, FGF21, and GDF‐15, were also considered as possible biomarkers and therapeutic targets in cancer cachexia (see review^128^) (*Table* *2*). The advantage of dosing muscle cytokines is that it is less invasive than collecting biopsy to evaluate muscle wasting. But more clinical data are needed to confirm this suggestion.

Data extracted from preclinical and clinical studies are also ambivalent. While most preclinical studies observed increased proteolysis and mitochondrial metabolism dysregulations in cachectic rodents, such alterations were not reported in numerous clinical studies on cancer cachexia. Petruzzelli and Wagner hypothesized that these differences were due to the severity of cachexia.^129^ Rodent studies are generally carried out during or after rapid and drastic weight loss, while muscle wasting is much more progressive in cachectic patients. The mismatch between the clinical reality and animal models could at least partly contribute to the poor translation observed in the field (see review^130^).

Even if there are discrepancies in the results of clinical studies, this review suggests the presence of defects in the structure of muscle fibres, increase in proteolysis, decrease in protein synthesis, alterations in lipid metabolism (myosteatosis), and dysfunctions in mitochondrial structure and metabolism (mitochondrial surface and dynamics and mitochondrial DNA) (*Table* *1*). Taken together, these observations suggest associations between structural and metabolic alterations that may, at least in part, be responsible for the skeletal muscle atrophy characteristics observed in cancer cachexia.

In addition to these parameters, it is interesting to note that endoplasmic reticulum (ER) stress has recently been studied in the context of cancer cachexia (see review^131^). An increase in the transcript levels (IRE1α, XPB‐1, ATF6, and DR5) and in protein levels (CHOP and phospho‐eIF2α) of certain ER stress markers has been reported in Lewis lung adenocarcinoma mice. These observations demonstrate that ER stress‐induced compensatory unfolding protein response (UPR) is increased in skeletal muscle from cachectic mice.^132^ Furthermore, this preclinical study suggests a close relationship between ER stress‐induced UPR and muscle proteolysis^132^ (see reviews^133^, ^134^). Furthermore, an increase in gene expression of ER stress‐induced UPR markers has recently been reported in the *vastus lateralis* muscle in cachectic patients with lung cancer.^135^ However, cancer cachectic patients were compared with healthy controls in this study.

**Figure 1:**
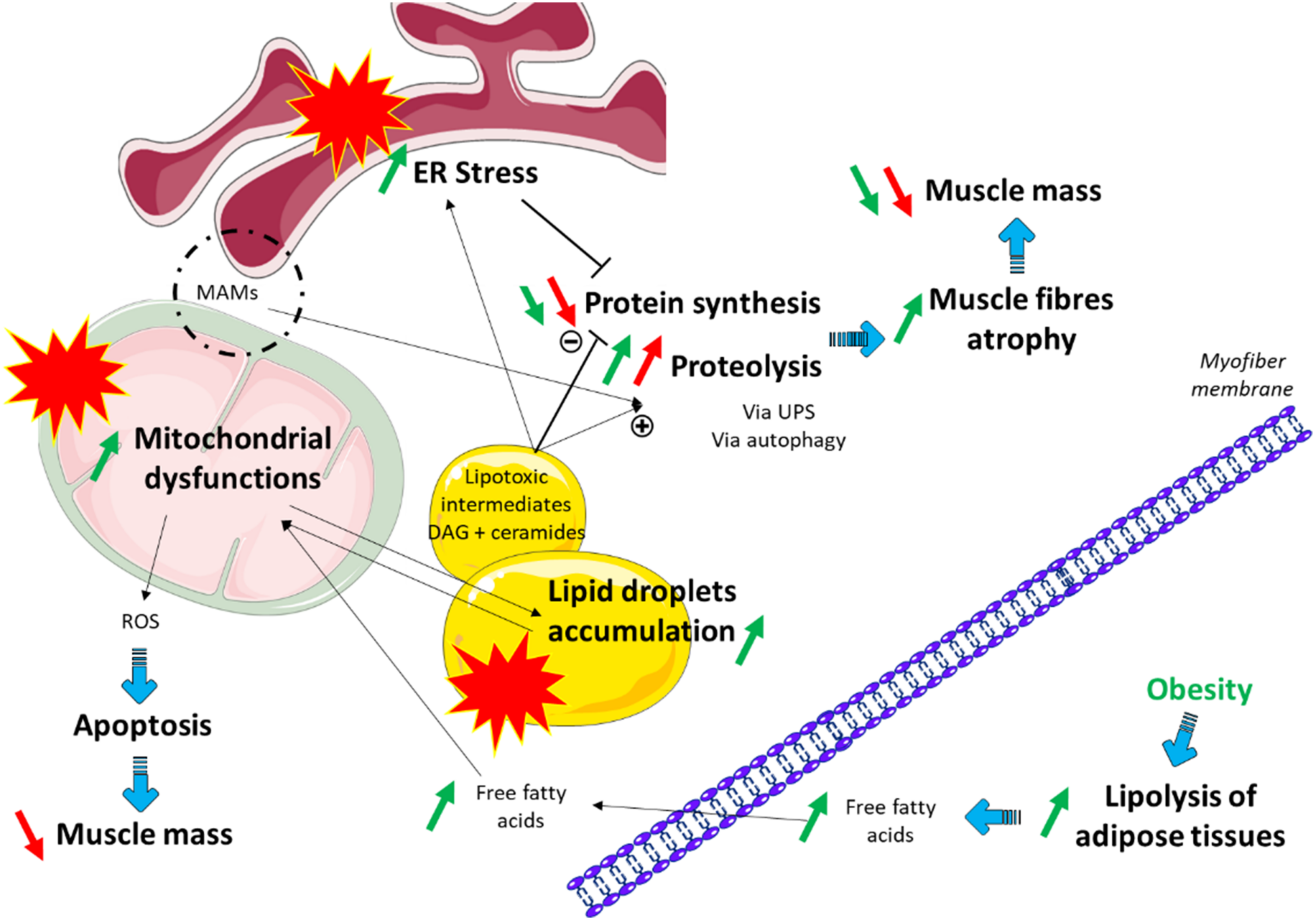
Summary of the hypothetical associations between metabolic and structural dysfunctions that may, at least in part, be responsible for skeletal muscle atrophy in cachectic cancer patients and for the aggravating role of obesity. In red are represented our hypotheses on the effects of cancer cachexia and in green, our hypotheses on the effects of obesity. An accumulation of lipid droplets, mitochondrial dysfunctions, and endoplasmic reticulum (ER) stress could lead to increased proteolysis, via the ubiquitin–proteasome system (UPS) and autophagy, and decreased protein synthesis, both of which induce atrophy of skeletal muscle fibres and loss of muscle mass. The production of reactive oxygen species (ROS) following mitochondrial alterations may also participate in muscle wasting, by activating apoptotic pathways. In the adipose compartment, lipolysis, which is increased in obese patients, could lead to a greater accumulation of lipid droplets in muscle fibres, mitochondrial dysfunctions, and disturbances in the integrity of mitochondria‐associated ER membranes (MAMs), which may worsen skeletal muscle atrophy.

How can the parameters cited previously be associated with muscle wasting? (*Figure* *1*) Myosteatosis is defined by lipid droplets accumulation in muscle. An accumulation of toxic lipid intermediates, such as ceramides and diacylglycerol, in lipid droplets may lead to increased proteolysis and decreased protein synthesis through inhibition of the Akt pathway^136^ (*Figure* *1*). These toxic lipid intermediates may also induce ER stress.^136^ To restore ER homeostasis, several triggers of the UPR may be activated. They include PERK, which may induce a decrease in protein synthesis through the ATF4 pathway.^137^ Intramuscular lipid accumulation may also cause dysfunctions in mitochondrial metabolism, through an inhibition of the electron transport chain and fatty acid β‐oxidation.^136^ Conversely, alterations in mitochondrial metabolism could limit fatty acid β‐oxidation, accentuate the accumulation of lipid droplets, and thereby create a vicious circle. Deterioration of mitochondrial metabolism may also actively participate in muscle wasting via the production of reactive oxygen species. Mitochondria and ER can interact via specific contact sites termed mitochondria‐associated ER membranes, which are necessary for several key processes, such as the formation of autophagosomes, the regulation of mitochondrial morphology, dynamics, quality control, calcium transport from the ER to the mitochondria, lipid synthesis and transport, proteolysis, as well as cell death.^138^, ^139^ Impairment of mitochondria‐associated membrane function could worsen dysfunctions in mitochondrial and lipid metabolisms and participate in the increase proteolysis, thereby inducing atrophy of skeletal muscle fibres and a decrease in skeletal muscle mass.

Skeletal muscle metabolism degradation during cancer cachexia has been poorly investigated in the context of obesity. Cancer‐induced skeletal muscle wasting and obesity present common skeletal muscle pathological mechanisms, such as insulin resistance, inflammatory state, and oxidative stress. Indeed, obesity predisposes to a pro‐inflammatory state via increased inflammatory mediators, such as TNF‐α and IL‐6, which stimulate the liver to synthesize and secrete C‐reactive protein. It is also associated with reduced levels of adiponectin, which has anti‐inflammatory function (see review^140^). This moderate inflammatory state might accelerate cancer‐induced cachexia. Effects of obesity on cancer cachexia are currently debated in the scientific community. While some scientists discuss the paradoxically protective effect of obesity,^141^ due to higher fat reserves, which would preserve from a deleterious weight loss, others highlight the danger of unseen muscle wasting.^142^ It has been suggested that the alterations of adipose tissue (when it occurs) precede muscle wasting in the development of cancer cachexia.^143^, ^144^ Adipose tissue secretes adipokines, especially in the context of obesity (see review^145^), which may have a direct effect on skeletal muscle metabolism: leptin, adiponectin, resistin, and visfatin are known mediators of tissue inflammation and insulin sensitivity (see reviews^146^, ^147^). Skeletal muscle may also release myokines, such as IL‐6 and IL‐15, which may increase white adipose tissue lipolysis/lipogenesis ratio.^128^, ^148^, ^149^ A vicious circle may exist between the increased adiposity and skeletal muscle wasting, especially because of the reciprocal influence exerted by these two tissues (see review^150^). For instance, adipose tissue lipolysis may induce myosteatosis. In obese patients, basal lipolysis has been reported to be increased in subcutaneous fat cells, because of their larger surface area associated with the secretion of inflammatory cytokines, such as TNF‐α.^151^ Excessive lipolysis leads to elevated fatty acid availability for skeletal muscle cells. Overloaded muscle fibres may present increased lipid droplet content (size and/or number), which leads to lipotoxic intermediate accumulation. In this context, muscle proteolysis, mitochondrial dysfunctions, and mitochondrial production of reactive oxygen species should be increased, which aggravates skeletal muscle wasting (*Figure* *1*). Potes *et al*. demonstrated that excess weight is associated with increased ER stress marker levels in human *vastus lateralis*.^152^ But further studies need to be performed in patients suffering from cancer cachexia to verify the presence or absence of ER stress and its interplay with the other molecular mechanisms involved in the onset and progression of cancer cachexia.

Similarly to this adipose tissue–muscle crosstalk, other tissues might affect the skeletal muscle: (i) liver acute‐phase proteins, such as serum amyloid A, could participate in activating muscle wasting by enhancing proteolysis (see review^153^); (ii) in the context of cancer with bone metastases, osteolysis induces the secretion of activin and TGF‐β, which are mediators of muscle proteolysis^154^; (iii) gut barrier dysfunction leads to increasing intestinal permeability of pathologic bacteria and endotoxemia and perpetuates systemic inflammation known to drive muscle wasting^155^; and (iv) interactions between systemic and brain inflammation^156^ alter the activity of the hypothalamus, responsible for the regulation of anorexia^157^ and for the secretion of glucocorticoids, which stimulate proteolysis in skeletal muscle.^158^

## Conclusion

The overall result of clinical studies suggests the presence of alterations in skeletal muscle from cachectic cancer patients, and these alterations could affect myofibers structure, proteolysis, and protein synthesis pathways, as well as lipid and mitochondrial metabolisms. However, evidences are still weak because clinical studies have often obtained contradictory results. Additional clinical studies are essential to further the exploring and understanding of alterations underlying skeletal muscle wasting characteristic of cancer cachexia. Moreover, an improvement and better standardization of the diagnostic criteria for cancer cachexia is crucial to ameliorate the robustness of the conclusions.

## Conflict of interest

The authors declare no conflict of interest.

## Funding

This work was partly supported by the ‘Ligue Nationale Contre le Cancer’ (comités 22, 29, 37, 85), by the ‘Institut National du Cancer’ (PLBio), and by the ‘Canceropole Grand Ouest’ (CONCERTO, Régions Centre‐Val de Loire and Pays de Loire et Bretagne). A.D. was supported by the Research French Ministry and the ‘Société Française de Nutrition Clinique et Métabolisme’ (SFNCM, Exceptional Research Prize).
